# Colour plasticity in response to social context and parasitic infection in a self-fertilizing fish

**DOI:** 10.1098/rsos.181418

**Published:** 2019-07-03

**Authors:** Rebecca Jane Pawluk, Carlos Garcia de Leaniz, Joanne Cable, Bernard Tiddeman, Sofia Consuegra

**Affiliations:** 1 Swansea University, Singleton Park, Swansea SA2 8PP, UK; 2 School of Biosciences, Cardiff University, Cardiff CF10 3AX, UK; 3 Department of Computer Science, Aberystwyth University, Aberystwyth SY23 3FL, UK

**Keywords:** colour plasticity, genotype, social, infection

## Abstract

Many animal species rely on changes in body coloration to signal social dominance, mating readiness and health status to conspecifics, which can in turn influence reproductive success, social dynamics and pathogen avoidance in natural populations. Such colour changes are thought to be controlled by genetic and environmental conditions, but their relative importance is difficult to measure in natural populations, where individual genetic variability complicates data interpretation. Here, we studied shifts in melanin-related body coloration in response to social context and parasitic infection in two naturally inbred lines of a self-fertilizing fish to disentangle the relative roles of genetic background and individual variation. We found that social context and parasitic infection had a significant effect on body coloration that varied between genetic lines, suggesting the existence of genotype by environment interactions. In addition, individual variation was also important for some of the colour attributes. We suggest that the genetic background drives colour plasticity and that this can maintain phenotypic variation in inbred lines, an adaptive mechanism that may be particularly important when genetic diversity is low.

## 1. Background

Animal coloration can indicate social status or the health condition of animals [1]. Colour signalling allows individuals to assess the social dominance, mating capability and/or health status of conspecifics without direct contact, and this can in turn influence reproductive success, social dynamics and the likelihood of becoming infected by directly transmitted parasites [2–4]. In vertebrates, changes in the distribution of melanin and carotenoids cause colour variation that has been related to behaviour, social dominance and infection status. Carotenoid pigments, responsible for bright orange coloration, have been widely studied for their role in the immune response of vertebrates and in the production of related signalling for sexual selection [5–7]. However, the signalling role of melanin is more controversial largely because, being endogenously produced, it does not seem to be as costly to produce or maintain as carotenoids, and because melanin-based coloration seems to be highly heritable [8]. Yet melanin-based coloration might also play a role in sexual selection through its link to body condition, as the genes involved in melanin production also regulate different phenotypic traits, which can be affected by frequency-dependent selection and/or local adaptation [9].

In vertebrates, changes in the distribution of melanin related to body colour variation have been linked with many physiological traits [5,10], including social dominance [11], stress responsiveness [12] and immune response to pathogens [13]. In a social context, teleost fish appear to use melanin for signalling subordination, which in brook trout (*Salvelinus fontinalis*) and tilapia (*Oreochromis niloticus*) occurs through darkening of the skin [14] or eye colour [15], respectively. These colour alterations in response to conspecifics may directly influence social structure, predation risk and population dynamics [16]. Individual colour may also be influenced by disease-causing agents; some teleost parasites can manipulate the host [17], potentially influencing colour and crypsis, in some cases, making infected individuals more vulnerable to predators [18]. For example, three-spine sticklebacks harbouring the parasitic worm *Schistocephalus solidius* show a gradual loss of colour in the skin and a darkening of the eye when compared with their healthy counterparts and this trend increases with increasing parasite size [2]. Such changes are caused by a decrease and/or redistribution of melanin in the skin of infected fish, making them less cryptic and more vulnerable to predator attacks [4]. Antibody production also correlates positively with the number of melanized spots in owl plumage [13]. Thus, there seems to be a link between changes in melanin coloration in relation to social and infection-related stress, but to what extent these responses are influenced by the genotype is unclear.

Body colour polymorphism has been observed among and within populations in many species, and its maintenance can have important evolutionary consequences [19]. Genetic polymorphisms in body colour can occur across populations as a result of advantageous heterozygosity, heterogeneous selection or frequency-dependent selection on rarer individuals, which can in turn result in trait variation among closely related individuals [20,21]. A number of individual genes have been identified to play a role in the regulation of melanin production (such as *Mc1r* in mammals and birds [22], and possibly in guppies [23], or *Oca2* in cave fish [24]), as well as QTLs [19,25], suggesting that the genetic basis of body coloration is complex and varies widely among taxa [17].

Colour polymorphisms can be important for the response of populations to variable selection pressures, such as parasite infections [17,26]. Furthermore, genetically diverse hosts tend to be less susceptible to parasitism than their less variable or inbred counterparts [26,27], and the interaction between host and parasite genotypes can elicit variable patterns of gene expression so that even genetically similar individuals can express varying gene shifts in response to infection [28]. For this reason, the extent to which genetic background determines phenotypic expression in response to parasites can be difficult to determine as individual variability complicates interpretation, particularly among vertebrates.

We have used a naturally inbred species, the mangrove killifish (*Kryptolebias marmoratus*), to investigate the importance of the genetic background in melanin-based colour change variation (plasticity) in response to parasitism and under different social contexts. This species is an ideal model for this study as its populations consist mainly of self-fertilizing, highly inbred hermaphrodites, which display low genetic diversity within selfing lines but are genetically different between lines [29–31]. *Kryptolebias marmoratus* are considered solitary and territorial [32] but have also been observed to congregate in crab burrows or inside logs [31,33] in high-density assemblages which can last for several months [34]. *Kryptolebias marmoratus* can display aggression towards conspecifics [29] and seem to prefer to associate with their kin [35]. Males also prefer the scent of hermaphrodites genetically different to them to those from the same selfing line [36]. Hermaphrodites have a mottled grey melanin-based colour with a black ocellus on the caudal peduncle and, although there is natural variation in individual colour, whether it varies in response to the social context and infection is not known [37]. The species also displays behavioural and transcriptomic variation in response to infection between genetically different lines [36,38]. On this basis, we hypothesized that, as for other teleosts, the social context and infection would result in colour changes in the hermaphrodites and that these differences would be strongly influenced by the genetic background of fish tested.

## 2. Methods

### 2.1. Experimental animals

We used two different *K. marmoratus* selfing lines (R and DAN) originating from wild populations in Belize, and subsequently bred in the laboratory for approximately 20–30 generations of selfing (lines were created in 2009 from 25 eggs from experimental lines maintained at the University of Guelph that had already undergone 10–20 generations of selfing [34]). Fish from the R strain are identical and homozygous at 28 of 29 microsatellite loci, while DAN fish form three distinct groups, varying from 27 to 29 homozygous loci [34]. All fish for the current study were age- and size-matched (within 1 mm) prior to testing and housed in individual tanks of approximately 7 × 7 × 6 cm where they were able to see other individuals but not smell or contact them. Water conditions for individuals were kept constant at 16 ppt salinity, 12 : 12 h light : dark photoperiod and 24°C.

### 2.2. Experiment 1: colour responses to social context

**Figure 1.**
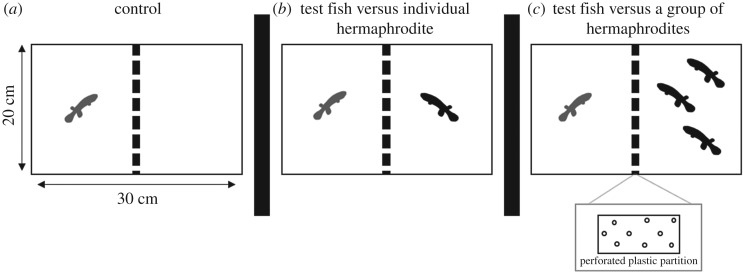
Schematic of experimental setting for social context experiments of *K. marmoratus*. (*a*) Control test fish set-up, (*b*) test fish facing a single hermaphrodite and (*c*) test fish facing a group of hermaphrodites.

The first experiment assessed colour change in response to different social situations (being housed with a single fish or with a group) within and between genotypic lines of *K. marmoratus*. Thirty-four mature, age-matched (between 22 and 26 months of age) hermaphroditic individuals were chosen as test fish from the two lines (17 DAN and 17 R). Three treatments were employed (figure 1): (i) control group where test fish were observed in a tank without any other fish (*n* = 8 per line); (ii) a single individual experiment (*n* = 9 per line); and (iii) a group experiment of three individuals (*n* = 9 per line). During the experiments, fish were allowed observation and scent smell but not physical contact. Prior to experimental testing, all fish were isolated whereby they could not see other fish. All fish were housed in individual plastic aquaria (12 × 8 × 8.5 cm, 16 ppt salinity, 24°C) for 11 days prior to the first trial. Test fish (aged between 24 and 36 months) were also isolated in the same manner (same aquaria and conditions) between social context treatment conditions (single or group context). All social context trials were conducted in aquaria (30 × 20 × 20.3 cm) divided into two equal parts by a transparent perforated partition allowing for visual and olfactory cues but eliminating physical contact between fish (figure 1). Test fish used to assess response to social context (nine DAN and nine R) were placed in one side of the tank and acclimated for 15 min prior to being tested against either a single hermaphroditic individual (single individual challenge) or a group of individuals (group context). Fish used to challenge the test fish (either single or as a group) were randomly chosen from a tank containing a mixture of R and DAN fish. Placement of tanks, line used and social context (single or group) were randomized using a random number generator and aquaria were thoroughly cleaned with ethanol and rinsed with distilled water between tests. After the first test, test fish were isolated for a further 11 days prior to being tested for the alternate condition. Photographs of test fish were taken on introduction of test fish and again at 24 and 48 h as described below.

### 2.3. Experiment 2: colour responses to infection

The second experiment assessed the extent of host colour change in response to infection by an ectoparasitic crustacean (*Argulus foliaceus*) as described previously [38]; this species is a generalist parasite that attaches by suction to the host skin, feeding continuously on blood and tissues. *Argulus foliaceus* causes wounding that induces innate and adaptive immune responses leading to reduced host fitness [39]. For this experiment, 80 mangrove killifish were used (40 DAN and 40 R); 20 fish of each line were infected with a single specimen of *A. foliaceus* for 48 h (providing enough time to trigger an adaptive immune response and the time at which 50% of the infected individuals had shed the parasite [38]), while the other 20 were kept as uninfected controls as described in [38]. *Argulus* successfully attached to all exposed fish. Fish were kept in individual aquaria (12 × 8 × 8.5 cm) containing 750 ml of water of diluted brackish water (14 ppt salinity, constituted from dechlorinated water and marine-filtered water, lowered from 16 ppt to increase parasite survival during the experiment) under the same light (12 : 12 h light: dark photoperiod) and temperature (24°C) conditions for the duration of the experiment. Upon infection, photographs were taken of each individual fish host immediately after infection, after 24 h and after 48 h (see below).

### 2.4. Image manipulation and colour value generation

Multiple images of all fish were taken using a Canon EOS 400D camera with a 18–55 mm EFS lens from a perpendicular distance of 30 cm to ensure a clear crisp image for analysis; the best image (JPEG) was selected for analysis. All photographs included an X-rite colour chart^®^ for calibration [40]. All images were calibrated prior to analysis using Adobe^®^ Lightroom^®^ Elements software and a profile created using the Xrite colour chart and Adobe^®^ DNG-profile editor^®^, to ensure light conditions were controlled. Each of the 240 photographs was then individually manipulated using the GNU Image Manipulation Program (GIMP) for use by the custom colour program. For each image, a mask was created of the whole fish that ensured the fish region was delimited and the background black. All images were run through a custom-made program (GetRegionColour; electronic supplementary material, table S1) to calculate average colour values over the selected region. All pixels in the mask region (grey value above 127 on 0–255 scale) were included in the average. Each pixel's RGB value was converted to the *XYZ* colour space and the CIE L*a*b* colour space (using the D65 illuminant as the reference white point) before averaging and the results were output to file. Values were represented in CIELAB space that uses a nonlinear transformation of the *XYZ* space to create L*, a* and b* values [40]. L* refers to lightness values from 0 (black) to 100 (absolute white), a* and b* are measures of colour on a 2D colour circle [41]; a* reflects the red/green colour scale and b* reflects the yellow/blue colour scale. To assess variations in chromatic attributes, a* and b* values were used to calculate hue (h*) for observable colour and chroma (C*) for colour saturation or brightness as described by van der Salm *et al*. [40]. Variation in light, hue and chroma colour attributes between time points (0–24, 0–48 and 24–48 h) was estimated for both treatment groups for statistical analysis.

### 2.5. Statistical analysis

Temporal shifts in colour attributes (i.e. before–after changes in lightness, hue and chroma) were examined in relation to parasitic infection, social context and genetic lines (R and DAN) using linear mixed effects models with the *lmer* function in the R package lme4 [41] using individual identity as random effects. Models with and without random factors were compared by the *anova* command and on the basis of AIC values by maximum likelihood (electronic supplementary material, table S2); models within 2 AIC units were considered equivalent [42] and the simplest of the two models was chosen; models were further simplified using step and drop1 functions for linear models and mixed effects models, respectively. Multiple comparisons were carried out using the *lsmeans* function in the R package lsmeans [43]. All analyses were run in R v. 3.4.0 [44].

## 3. Results

### 3.1. Colour shifts in response to social context

**Figure 2.**
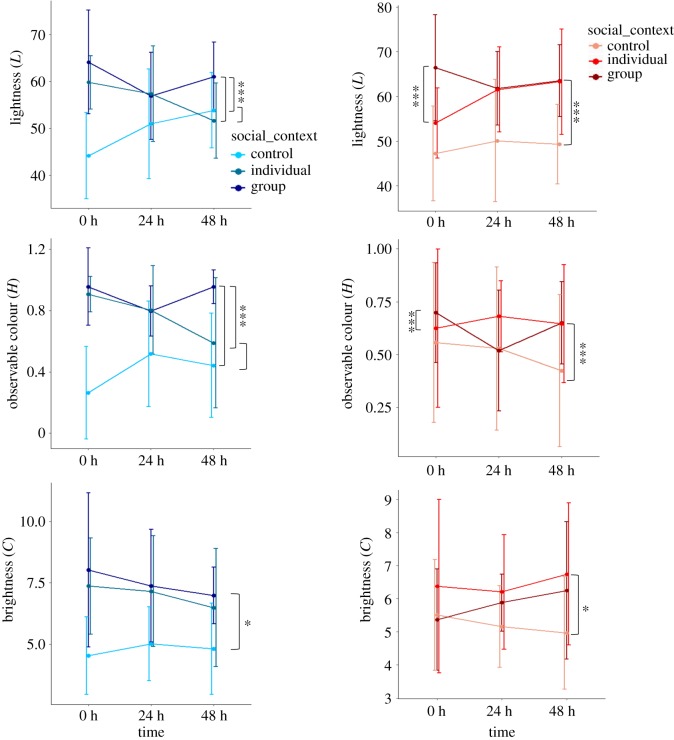
Variations in light (*L*), hue (*H*) and chroma (*C*) between lines (on the left, DAN in blue, and on the right, R in red; controls are represented in grey). These were compared with respect to social grouping over time for 16 control individuals (8 DAN and 8 R) and 18 test individuals (9 DAN and 9 R). Significant comparisons between groups are indicated by an asterisk (*p < 0.05, ***p < 0.001).

Changes in fish lightness, hue and chroma values were compared between control, single individual tests and group contexts for all time periods (table 1 and figure 2). A comparison among four models including and excluding interactions between line, time and social context, two of them including individual (ID) as random factor, was conducted for each colour attribute (electronic supplementary material, table S2). For light and chroma, the model which included social context, line, time and ID (without interactions) provided the best fit to the data when all individuals were considered, and after simplification these models only included social context and ID. For hue, the model which included social context, line and time (with interactions) provided the best fit to the data when all individuals were considered (electronic supplementary material, table S2). Variation in fish colour attributes did not differ significantly between fish exposed to individuals or groups of fish (light: *t*_15.998_ = −0.766, *p* = 0.455, hue: *t*_11.785_ = 0.099, *p* = 0.923, chroma: *t*_11.616_ = 1.369, *p* = 0.197). However, changes in body lightness differed significantly depending on social context when compared with controls (*t*_93.85_ = 5.066, *p* < 0.001, table 1); fish paired with individuals or social groups appeared lighter than controls and those paired with a group appeared lighter than those paired with an individual (post hoc tests; electronic supplementary material, table S2a). There was also an effect of individual identity on changes in lightness when comparing models (*χ*^2^ = 22.45, d.f. = 2, *p* < 0.001; electronic supplementary material, table S2a). Changes in hue also differed significantly with social context (*t*_238_ = 5.852, *p* < 0.001). Additionally, there was a significant interaction between line and social context on changes in hue (*t*_236_ = −3.498, *p* ≤ 0.001). As with light, line and time had no significant influence on temporal variation in hue (*t*_238_ = 1.458, *p* = 0.147). There was no effect of individual identity on colour shifts and none of the other interactions were significant (table 1).

**Table 1.** Effects of genetic line, social context and time on three colour attributes. Significant differences are indicated by asterisks.

colour attribute and predictor	estimate	std. error	d.f.	*t*-value	*p*-value
lightness					
social context	5.55416	1.09641	238	5.066	<0.000***
hue					
line	0.111	0.0765	238	1.458	0.146
social context	0.244	0.0411	238	5.952	<0.000***
line : social context	−0.205	0.0587	236	−3.529	<0.000***
chroma					
social context	0.486730	0.224324	53.65	2.170	0.034*

Social context significantly influenced change in chroma (brightness of fish); fish became much brighter when they paired with other individuals than unpaired controls (*t*_109.25_ = 2.170, *p* = 0.03), and those paired with a group of fish became brighter than those paired with an individual fish (electronic supplementary material, table S2c). Shifts in chroma were, however, influenced by individual identity when models with and without ID as a factor were compared (*χ*^2^ = 25.47, d.f. = 2, *p* < 0.001; electronic supplementary material, table S2c).

### 3.2. Colour shifts in response to infection

**Figure 3.**
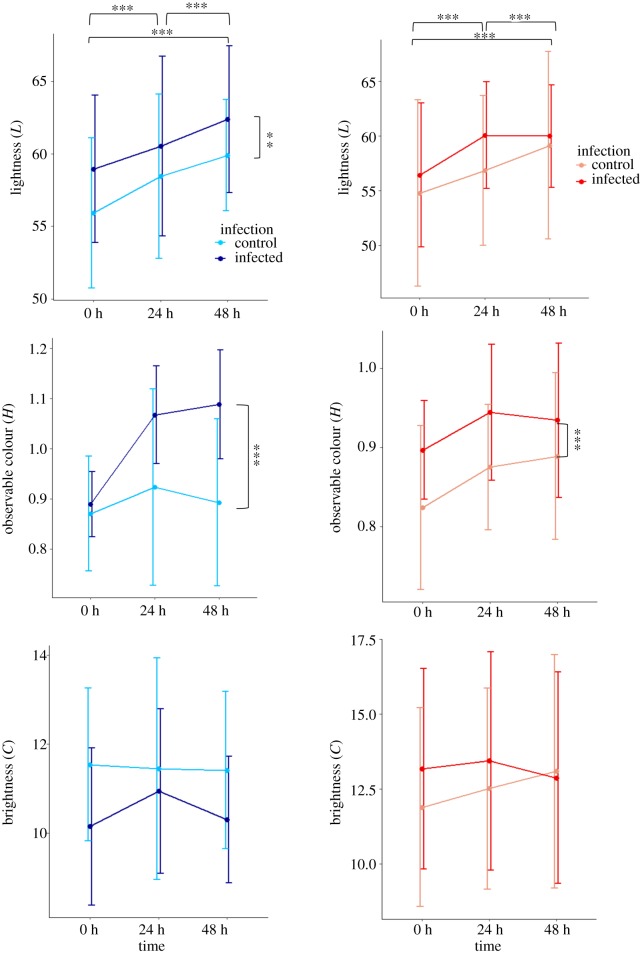
Variations in light (*L*), hue (*H*) and chroma (*C*) between lines in response to infection over time for 20 infected and 20 control individuals for both lines (on the left, DAN in blue and on the right, R in red, controls are represented in grey).

Changes in light, hue and chroma values were compared between treatments and for all time periods for 40 R and 40 DAN individuals, of which 20 were controls and 20 infected fish from each line (figure 3 and table 2). Comparisons were made with and without interactions, and with and without random factors (electronic supplementary material, table S3). For light and hue values, the most plausible model included infection treatment (infected versus control), line and time (without ID as a factor); the hue model included interactions between factors (electronic supplementary material, table S3a,b,). For chroma, the most plausible model included infection treatment (infected versus control), line, time and ID (without interactions) when all individuals were considered (electronic supplementary material, table S3c); after simplification, the best model included only line and ID.

**Table 2.** Effects of genetic line, infection status and time on colour shifts of mangrove killifish experimentally infected with *Argulus*. Significant differences are indicated by asterisks.

colour attribute and predictors	estimate	std. error	d.f.	*t*-value	*p*-value
lightness					
line	−1.48398	0.77553	25.37	−1.913	0.056
infection	2.21344	0.77553	93.85	2.854	0.005**
time	0.07976	0.01979	119.34	4.031	<0.001***
hue					
line	−0.0327020	0.0208995	148	−1.565	0.119
infection	0.1197282	0.0208995	148	5.729	<0.001***
time	0.0004664	0.0007541	148	0.619	0.536
line : infection	−0.0573730	0.0295564	148	−1.941	0.053
line : time	0.0008903	0.0010665	148	0.835	0.404
infection : time	0.0036866	0.0010665	148	3.457	<0.001***
line : infection : time	−0.0042634	0.0015083	148	−2.827	0.005**
chroma					
line	1.856607	0.593470	28.05	3.128	0.003**

Changes in body lightness differed significantly between infection status (*t*_238_ = 2.854, *p* = 0.005) and length of time infected (*t*_238_ = 4.031, *p* ≤ 0.001); infected individuals became lighter than controls, and lightness increased with the length of time the fish were infected. Shifts in lightness were not influenced by line (*t*_238_ = −1.913, *p* = 0.056). In contrast with social context, changes in skin lightness were not influenced by individual variation (table 2; electronic supplementary material, table S3). Individual identity had no effect on hue (observable colour) (electronic supplementary material, table S3b); however, shifts in hue were significantly influenced by infection status (*t*_238_ = 5.729, *p* < 0.001), the interaction between infection status and time (*t*_236_ = 3.457, *p* = 0.001) and the interaction between all factors (*t*_238_ = −2.827, *p* = 0.005). Analysis of significant interactions indicated that changes in hue were higher in infected fish than controls; an increase in hue occurred over time and it was always higher in the infected group. Similarly, changes in chroma (brightness) differed significantly between lines (*t*_54.49_ = 3.128, *p* = 0.003) being higher in R than DAN individuals; this attribute was also influenced by individual identity when models were compared (*χ*^2^ = 197.41*,* d.f. = 2, *p* < 0.001; electronic supplementary material, table S3c).

## 4. Discussion

Environmental fluctuations, such as the presence of conspecifics or parasitic infection, have the potential to influence the phenotypic traits exhibited by individuals, including colour. Our results suggest that both infection and social context influence lightness, observable colour and brightness in an inbred fish species which, to some extent, also displayed individual variation. Specific responses to environmental fluctuations can be difficult to identify in natural populations due to the high degree of individual genetic variation present [45]. Using a naturally inbred species allowed for estimation of the influence of the genotype on melanin-based coloration as well as the overall physiological colour change response to treatments under controlled rearing conditions.

Alterations in social grouping or novel threats from conspecifics have been shown to alter melanophore distribution, the extent of observable colour displayed and brightness attributes in a variety of species from across the Animal Kingdom [2,5,46,47]. Here, we showed that fish faced with small social groups became lighter than control fish faced with blank water, and the number of individuals in the group further influenced the individuals' colour. In teleost species, changes in dark pigmentation (melanization or de-melanization) of the skin of an individual are commonly used as an indicator of social status, for example, juvenile Atlantic salmon and Arctic charr display darker pigmentation as a signal of submission to opponents [5]. Phenotypic alterations as a means of signalling to conspecifics allow a quick approximation of status within groups. Mangrove killifish have previously shown to be aggressive towards their conspecifics [29,48], particularly when they are unrelated [33], and the level of aggression has been related to individual cortisol and testosterone levels [48]. As melanin-based coloration has been related to hormone levels [8], the observed lightening of skin colour in killifish faced with social groups could indicate dominance [11,12].

Similar to social context, parasitized fish became lighter in skin coloration compared to controls, and the longer the time of infection, the lighter the fish became. These results could indicate a potentially similar de-melanization effect in parasitized killifish as seen in *Schistocephalus solidus-*infected sticklebacks [2]. These results also support a link between physiological body condition and melanin-based colour; in wild populations, lighting of skin colour may influence predator–prey dynamics [17] whereby infected killifish would be less cryptically coloured in their environment in a similar way to *Diplostomum spathaceum*-infected rainbow trout [18]. It is also plausible that de-melanization could be used by killifish as a form of signalling, similar to the way in which colour is used as an honest signal in turtles [7]. Further to this, observable colour (hue) and brightness (chroma) were also affected by infection status. Observable colour increases rapidly in response to stress in the red porgy, *Pagrus pagrus* [41], while brightness decreases in parasitized guppies [47], supporting the hypothesis that the changes in colour observed here could be due to stress caused by social context or infection. These colour changes could potentially influence the way in which hermaphroditic individuals are perceived, an important factor for this facultative selfing species, where outcrossing is limited and possibly driven by males [34]. If colour was important for mating decisions, it could also influence genetic variation within populations and, consequently, the ability to respond to environmental fluctuations [35]. Although we cannot completely discard that the observed changes could be influenced by experimental conditions, which could create stress independently from the infection or social stress, the differences observed between the infected and control group (which was subject to the same experimental stress and mock infection) and between the different social tests suggest that the colour variation was due to both infection and social context, respectively.

Alterations in lightness of individuals were not affected by genotype in any of the experimental conditions, suggesting a degree of plasticity for this colour attribute. Conversely, genetic line influenced changes in observable colour and brightness in both experimental tests, with an interaction between genotype and the social environment, which could suggest the existence of genotype by environment interactions [48]. Yet our results also highlighted the plasticity of individual variation in colour responses. This suggests that other individual factors, apart from genotypic variation, could also drive variation in colour changes in relation to social context and parasitic infection [49]. Individual flexibility in phenotypic (colour) response can be key for indicating health, dominance or mating.

In summary, our results indicate that melanin-based colour can change relatively quickly in the mangrove killifish in response to infection or social interactions, and that change might act as a proxy for body condition, in the mangrove killifish as suggested for other species [9]. Thus, in infected individuals, colour could be indicative of mate health status if/when hermaphroditic individuals come into contact with males. If the variation observed between selfing lines in colour in response to social context and parasitic infection was the result of genotype by environment interactions, it may be particularly important to maintain colour polymorphism in this inbred species with very limited genetic diversity.

## Supplementary Material

Supplementary material

## Data accessibility

Data available from the Dryad Digital Repository at: https://doi.org/10.5061/dryad.t7p41f4 [50].
