# Supplementary material for: Colour plasticity in response to social context and parasitic infection in a self-fertilizing fish
Source: R Soc Open Sci. 2019 Jul 3;6(7):181418. doi: 10.1098/rsos.181418 (PMC6689574; doi:10.1098/rsos.181418)
Supplement: Supplementary material [file rsos181418supp1.docx]

**Supplementary material for:** **Colour plasticity in response to social context and parasitic infection in a self-fertilizing fish**

Rebecca Jane Pawluk, Carlos Garcia de Leaniz, Jo Cable, Bernard Tiddeman, Sofia Consuegra

**Table S1**. Algorithms for colour conversion. The conversion from *sRGB* colour space to the *CIE XYZ* colour space is given by a non-linear conversion:

for *k* = *r*, *g* and *b* (to give r’, g’ and b’)

$k'=\left\{ \begin{matrix} \left( \frac{(k+0.055)}{1.055} \right)^{2.4} & \text{if} k>0.04045 \\ \frac{k}{12.92} & \text{otherwise} \end{matrix} \right.$

followed by a linear transformation:

$\left( \begin{matrix} X \\ Y \\ Z \end{matrix} \right)=\left( \begin{matrix} 0.4124 & 0.3576 & 0.1805 \\ 0.2126 & 0.7152 & 0.0722 \\ 0.0193 & 0.1192 & 0.9505 \end{matrix} \right)\left( \begin{matrix} r' \\ g' \\ b' \end{matrix} \right)$

The formulae for converting from XYZ to L*a*b* are given by:

*f_0_* = *f*(*y/w_y_*)

*L** = *116*f_0_-16*

*a** = *500**(*f*(*x*/*w_x_*)-*f_0_*)

*b** = *200**(*f_0_-f(z*/*w_z_))*

Where the *XYZ* colour is represented by (*x, y, z*), and the whitepoint is (*w_x_, w_y_, w_z_*). The function *f* is defined as:

$f\left( x \right)=\left\{ \begin{matrix} \sqrt[3]{x} & \text{if} x>\delta^{3} \\ \frac{x}{3\delta^{2}}+\frac{4}{29} & \text{otherwise} \end{matrix} \right.$

where δ = 6/29.

The source code implementing the above is available at <https://github.com/bptiddeman/get-region-colour>.

**Table S2**. Comparison among models of colour expression attributes (Light, hue, chroma) in *Kryptolebias marmoratus* as a function of social context (single, group or control) and selfing line (DAN and R), including and excluding individual (ID) as a random factor. (a) Lightness values (b) Hue values (c) Chroma values. Post-hoc testing was completed using lsmeans (package lsmeans) on the final selected model for each attribute.

**a) Model comparison**:

modL3: L ~ Line + Social + Time

modL1: L ~ Line + Social + Time + (-1 + isDAN | ID) + (1 + isR | ID)

modL4: L ~ Line * Social * Time

modL2: L ~ Line * Social * Time + (-1 + isDAN | ID) + (1 + isR | ID)

Df AIC BIC logLik deviance Chisq Chi Df Pr(>Chisq)

modL3 5 1162.1 1177.3 -576.04 1152.1

modL1 7 1143.6 1165.0 -564.81 1129.6 22.458 2 1.328e-05 ***

modL4 9 1164.1 1191.5 -573.04 1146.1 0.000 2 1

modL2 11 1144.4 1177.9 -561.19 1122.4 23.687 2 7.185e-06 ***

**Posthoc tests**:

Social lsmean SE df lower.CL upper.CL

0 50.61784 1.571765 50.04 47.46093 53.77475

1 56.17200 1.245165 25.12 53.67107 58.67293

2 61.72615 1.742025 43.52 58.22727 65.22504

contrast estimate SE df t.ratio p.value

0 - 1 -5.554156 1.096410 93.85 -5.066 <.0001

0 - 2 -11.108311 2.192819 93.85 -5.066 <.0001

1 - 2 -5.554156 1.096410 93.85 -5.066 <.0001

Models:

modL3: L ~ Line + Social + Time

modL1: L ~ Line + Social + Time + (-1 + isDAN | ID) + (-1 + isR | ID)

Df AIC BIC logLik deviance Chisq Chi Df Pr(>Chisq)

modL3 5 1162.1 1177.3 -576.04 1152.1

modL1 7 1143.6 1165.0 -564.81 1129.6 22.458 2 1.328e-05 ***

**Model simplification:**

Formula: L ~ Social + (-1 + isDAN | ID) + (-1 + isR | ID)

Data: Llmm.data

Scaled residuals:

Min 1Q Median 3Q Max

-2.48532 -0.49937 0.03257 0.53356 2.74019

Random effects:

Groups Name Variance Std.Dev.

ID isDAN 16.23 4.029

ID.1 isR 55.44 7.446

Residual 64.60 8.038

Number of obs: 156, groups: ID, 34

Fixed effects:

Estimate Std. Error df t value Pr(>|t|)

(Intercept) 50.358 1.493 57.259 33.737 < 2e-16 ***

Social 5.546 1.089 94.864 5.091 1.8e-06 ***

**b) Model comparison**:

modL3: H ~ Line + Social + Time

modL1: H ~ Line + Social + Time + (-1 + isDAN | ID) + (1 + isR | ID)

modL4: H ~ Line * Social * Time

modL2: H ~ Line * Social * Time + (-1 + isDAN | ID) + (1 + isR | ID)

Df AIC BIC logLik deviance Chisq Chi Df Pr(>Chisq)

modL3 5 78.204 93.453 -34.102 68.204

modL1 9 74.197 101.646 -28.099 56.197 12.0065 4 0.01730 *

modL4 9 72.817 100.266 -27.409 54.817 1.3800 0 < 2e-16 ***

modL2 13 72.253 111.901 -23.126 46.253 8.5642 4 0.07297 .

**c) Model comparison**:

M3_C: C ~ Line + Social + Time

M1_C: C ~ Line + Social + Time + (-1 + isDAN | ID) + (1 + isR | ID)

M4_C: C ~ Line * Social * Time

M2_C: C ~ Line * Social * Time + (-1 + isDAN | ID) + (1 + isR | ID)

Df AIC BIC logLik deviance Chisq Chi Df Pr(>Chisq)

M3_C 5 662.06 677.31 -326.03 652.06

M1_C 7 640.59 661.94 -313.30 626.59 25.470 2 2.947e-06 ***

M4_C 9 659.92 687.37 -320.96 641.92 0.000 2 1

M2_C 11 638.54 672.09 -308.27 616.54 25.383 2 3.078e-06 ***

**Posthoc tests**:

Social lsmean SE df lower.CL upper.CL

0 5.531723 0.3166044 53.39 4.896803 6.166643

1 6.018453 0.2509449 27.73 5.515207 6.521699

2 6.505184 0.3554585 47.60 5.792346 7.218022

contrast estimate SE df t.ratio p.value

0 - 1 -0.4867305 0.2243239 109.25 -2.17 0.0810

0 - 2 -0.9734609 0.4486478 109.25 -2.17 0.0810

1 - 2 -0.4867305 0.2243239 109.25 -2.17 0.0810

Models:

M3_C: C ~ Line + Social + Time

M1_C: C ~ Line + Social + Time + (-1 + isDAN | ID) + (-1 + isR | ID)

Df AIC BIC logLik deviance Chisq Chi Df Pr(>Chisq)

M3_C 5 662.06 677.31 -326.03 652.06

M1_C 7 640.59 661.94 -313.30 626.59 25.47 2 2.947e-06 ***

**Model simplification:**

Formula: C ~ Social + (-1 + isDAN | ID) + (-1 + isR | ID)

Data: Clmm.data

Scaled residuals:

Min 1Q Median 3Q Max

-2.2029 -0.6758 -0.1060 0.5560 2.4078

Random effects:

Groups Name Variance Std.Dev.

ID isDAN 1.986 1.409

ID.1 isR 1.069 1.034

Residual 2.512 1.585

Number of obs: 156, groups: ID, 34

Fixed effects:

Estimate Std. Error df t value Pr(>|t|)

(Intercept) 5.4874 0.3125 58.8673 17.560 <2e-16 ***

Social 0.4796 0.2239 112.2592 2.142 0.0343 *

**Table S3**. Among model comparison of colour expression attributes (Light, Hue, Chroma) in *Kryptolebias marmoratus* as a function of infection treatment (infection or control) and selfing line (DAN and R), including and excluding individual (ID) as a random factor. (a) Lightness values (b) Hue values (c) Chroma values. Post-hoc testing was completed using lsmeans (package lsmeans) on the final selected model for each attribute.

**a) Model comparison**:

Models:

modL3: L ~ Line + Infection + Time

modL1: L ~ Line + Infection + Time + (-1 + isDAN | ID) + (1 + isR | ID)

modL4: L ~ Line * Infection * Time

modL2: L ~ Line * Infection * Time + (-1 + isDAN | ID) + (1 + isR | ID)

Df AIC BIC logLik deviance Chisq Chi Df Pr(>Chisq)

modL3 5 1547.7 1565.1 -768.84 1537.7

modL1 9 1555.2 1586.5 -768.58 1537.2 0.5310 4 0.9704

modL4 9 1555.4 1586.7 -768.68 1537.4 0.0000 0 1.0000

modL2 13 1562.8 1608.1 -768.42 1536.8 0.5262 4 0.9709

**Posthoc tests**:

**Infection** lsmean SE df lower.CL upper.CL

0 57.51551 0.548385 236 56.43516 58.59587

1 59.72896 0.548385 236 58.64860 60.80931

contrast estimate SE df t.ratio p.value

0 - 1 -2.213444 0.7755335 236 -2.854 0.0047

**Time**  lsmean SE df lower.CL upper.CL

0 56.70798 0.6131131 236 55.50011 57.91586

24 58.62224 0.3877668 236 57.85831 59.38616

48 60.53649 0.6131131 236 59.32861 61.74436

contrast estimate SE df t.ratio p.value

0 - 24 -1.914253 0.4749154 236 -4.031 0.0002

0 - 48 -3.828506 0.9498307 236 -4.031 0.0002

24 - 48 -1.914253 0.4749154 236 -4.031 0.0002

**b) Model comparison**:

modL3: H ~ Line + Infection + Time

modL1: H ~ Line + Infection + Time + (-1 + isDAN | ID) + (1 + isR | ID)

modL4: H ~ Line * Infection * Time

modL2: H ~ Line * Infection * Time + (-1 + isDAN | ID) + (1 + isR | ID)

Df AIC BIC logLik deviance Chisq Chi Df Pr(>Chisq)

modL3 5 -338.79 -321.39 174.40 -348.79

modL1 9 -342.48 -311.15 180.24 -360.48 11.6881 4 0.019828 *

modL4 9 -349.41 -318.09 183.71 -367.41 6.9342 0 < 2.2e-16 ***

modL2 13 -355.22 -309.97 190.61 -381.22 13.8047 4 0.007945 **

**c) Model comparison**:

Models:

modL3: C ~ Line + Infection + Time

modL1: C ~ Line + Infection + Time + (-1 + isDAN | ID) + (1 + isR | ID)

modL4: C ~ Line * Infection * Time

modL2: C ~ Line * Infection * Time + (-1 + isDAN | ID) + (1 + isR | ID)

Df AIC BIC logLik deviance Chisq Chi Df Pr(>Chisq)

M3_C 5 1184.72 1202.1 -587.36 1174.72

M1_C 9 995.31 1026.6 -488.66 977.31 197.41 4 <2e-16 ***

M4_C 9 1185.56 1216.9 -583.78 1167.56 0.00 0 1

M2_C 13 992.63 1037.9 -483.31 966.63 200.93 4 <2e-16 ***

**Posthoc tests**:

Line lsmean SE df lower.CL upper.CL

DAN 10.96455 0.2504377 37.74 10.45745 11.47164

R 12.82115 0.5380402 38.39 11.73170 13.91060

contrast estimate SE df t.ratio p.value

DAN - R -1.856607 0.5934698 54.49 -3.128 0.0028

Models:

M3_C: C ~ Line + Infection + Time

M1_C: C ~ Line + Infection + Time + (-1 + isDAN | ID) + (-1 + isR |

M1_C: ID)

Df AIC BIC logLik deviance Chisq Chi Df Pr(>Chisq)

M3_C 5 1184.72 1202.1 -587.36 1174.72

M1_C 7 991.31 1015.7 -488.66 977.31 197.41 2 < 2.2e-16 ***

**Model simplification:**

Formula: C ~ Line + (-1 + isDAN | ID) + (-1 + isR | ID)

Data: Clmm.data

Scaled residuals:

Min 1Q Median 3Q Max

-2.94096 -0.60054 -0.03657 0.54735 3.00698

Random effects:

Groups Name Variance Std.Dev.

ID isDAN 2.149 1.466

ID.1 isR 10.616 3.258

Residual 1.628 1.276

Number of obs: 240, groups: ID, 80

Fixed effects:

Estimate Std. Error df t value Pr(>|t|)

(Intercept) 10.9645 0.2594 39.0000 42.267 < 2e-16 ***

LineR 1.8566 0.5884 56.7818 3.155 0.00257 *

**Figure S1**. Fish ID R_14 at the start of the treatment (0h, a) and after 48 h of infection (b)

**
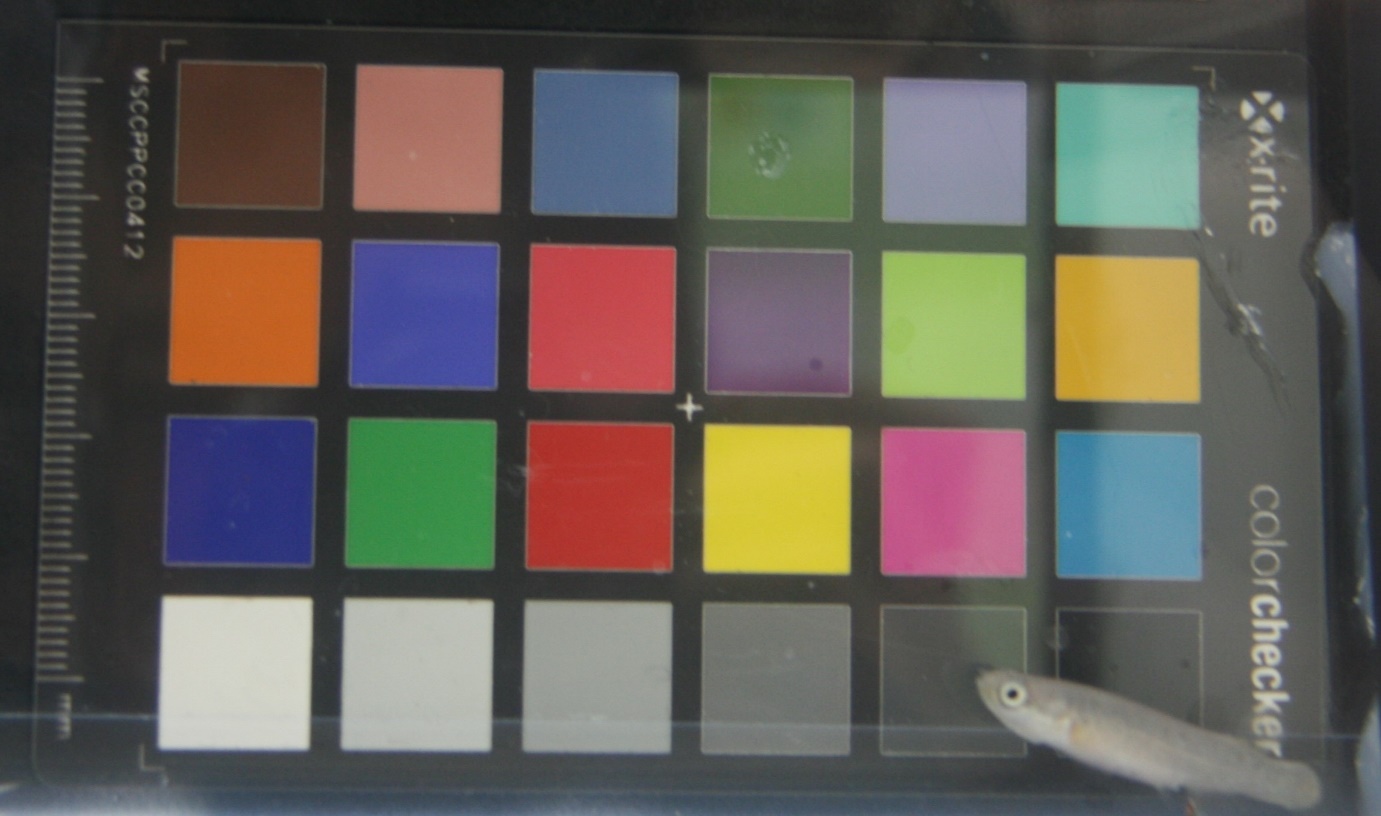
(a)**

**
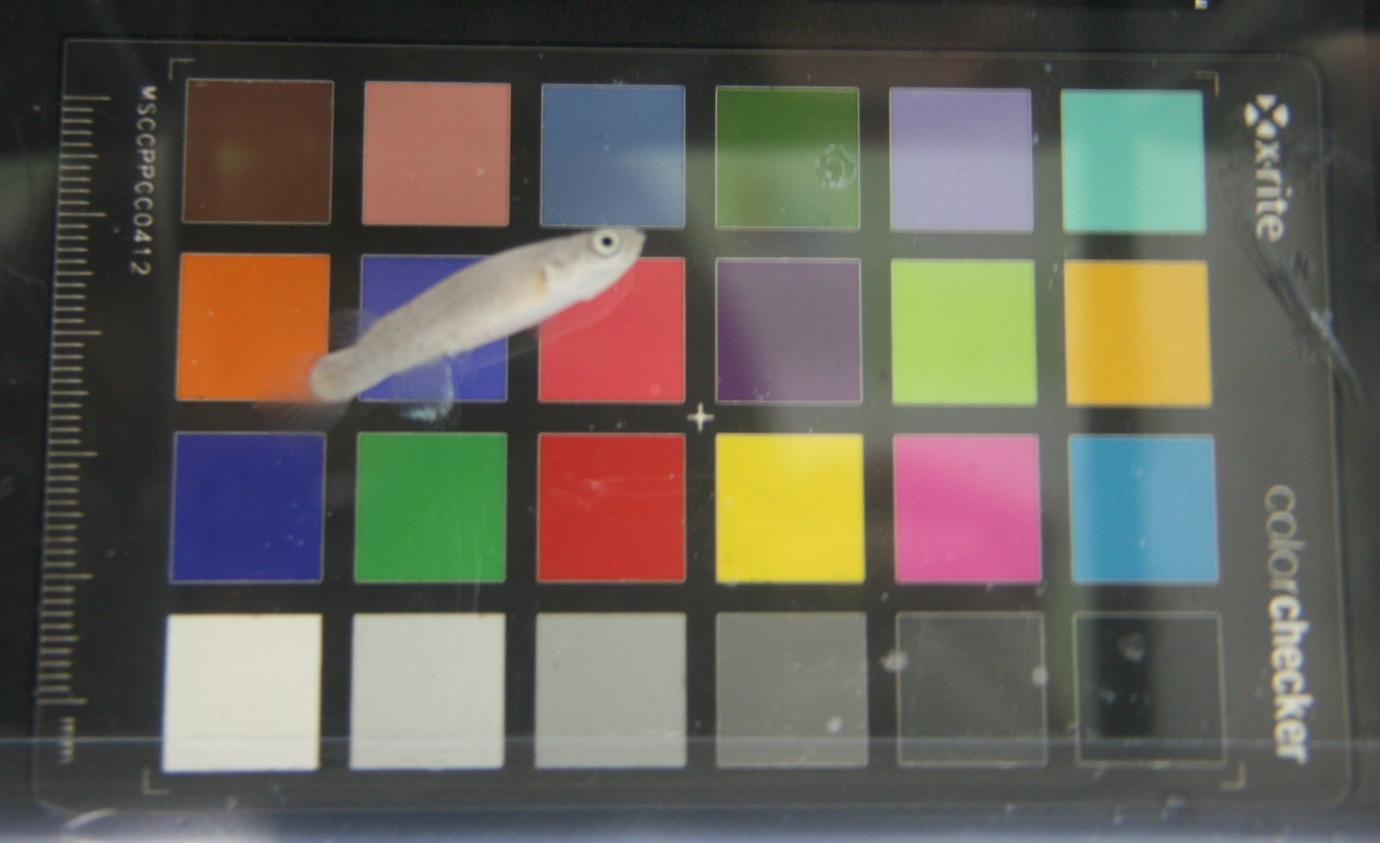
(b)**
